# Autophagy machinery plays an essential role in traumatic brain injury-induced apoptosis and its related behavioral abnormalities in mice: focus on *Boswellia* Sacra gum resin

**DOI:** 10.3389/fphys.2023.1320960

**Published:** 2024-01-05

**Authors:** Livia Interdonato, Ylenia Marino, Daniela Impellizzeri, Ramona D’Amico, Rosalba Siracusa, Roberta Fusco, Gaetano Cammilleri, Licia Pantano, Sergio Modafferi, Ali S. Abdelhameed, Tilman Fritsch, Luay J. Rashan, Salvatore Cuzzocrea, Vittorio Calabrese, Marika Cordaro, Rosanna Di Paola

**Affiliations:** ^1^ Department of Chemical and Biological, Pharmaceutical and Environmental Sciences, University of Messina, Messina, Italy; ^2^ Chemistry Department, Istituto Zooprofilattico Sperimentale Della Sicilia, Palermo, Italy; ^3^ Department of Biomedical and Biotechnological Sciences, University of Catania, Catania, Italy; ^4^ Department of Pharmaceutical Chemistry, College of Pharmacy, King Saud University, Riyadh, Saudi Arabia; ^5^ NAM-Institute, Salzburg, Austria; ^6^ Medicinal Plants Division, Research Center, Dhofar University, Salalah, Oman; ^7^ Department of Biomedical, Dental and Morphological and Functional Imaging University of Messina, Messina, Italy; ^8^ Department of Veterinary Sciences, University of Messina, Messina, Italy

**Keywords:** TBI, autophagy, apoptosis, behavioral, *Boswellia* sacra

## Abstract

Traumatic brain injury (TBI) is described as a structural damage or physiological disturbance of brain function that occurs after trauma and causes disability or death in people of all ages. New treatment targets for TBI are being explored because current medicines are frequently ineffectual and poorly tolerated. There is increasing evidence that following TBI, there are widespread changes in autophagy-related proteins in both experimental and clinical settings. The current study investigated if Boswellia Sacra Gum Resin (BSR) treatment (500 mg/kg) could modulate post-TBI neuronal autophagy and protein expression, as well as whether BSR could markedly improve functional recovery in a mouse model of TBI. Taken together our results shows for the first time that BSR limits histological alteration, lipid peroxidation, antioxidant, cytokines release and autophagic flux alteration induced by TBI.

## 1 Introduction

With more than 1.7 million new cases each year and 60% of all trauma-related deaths in the U.S., TBI is a significant public health issue. TBI causes secondary brain injury, which sets off a chain reaction of pathophysiological events that cause neuronal cell death, brain edema, and neurological impairments. These events include oxidative stress, autophagy, inflammation, and apoptosis. However, there are currently no viable treatments for TBI patients undergoing clinical intervention. Understanding the pathophysiological mechanisms following TBI and locating new therapeutic methods are thus crucial and urgent (Zeng et al., 2020). The latter indicates delayed and perhaps reversible molecular and cellular pathophysiological pathways that start shortly after the first injury and may last for months or years (Bramlett and Dietrich, 2007; Wu and Lipinski, 2019). Despite the fact that most current research has focused on the earliest cellular and molecular events, experimental and clinical data indicate that central nervous system (CNS) trauma-mediated pathophysiological changes may persist for years, causing chronic post-mitotic cell loss and activation of microglia and astrocytes as well as chronic functional deficits (Ramlackhansingh et al., 2011). A growing database of research shows that substantial changes in autophagy-related proteins occur after TBI in both experimental and clinical settings (Zeng et al., 2020). Neurological impairments and mortality are mostly caused by cell death following neurotrauma. Even though CNS damage affects many different cell types, including neurons and oligodendrocytes, the mechanisms of neuronal cell death have received most of the attention. Multiple cell death mechanisms exist in the damaged CNS after trauma such as apoptosis and autophagy (Stoica and Faden, 2010; Schoch et al., 2012). Long-lived cytosolic proteins and damaged organelles increase a defective autophagic machinery that could lead to apoptosis. The transfer of the desired components to the lysosome includes a series of sequential steps, including the creation of a double membrane, elongation, and ultimately vesicle maturation. The morphology of apoptotic cell is the best way to explain it. Cell rounding, membrane blebbing, cytoskeletal collapse, cytoplasmic condensation and fragmentation, nuclear pyknosis, chromatin condensation and fragmentation, and the development of membrane-encased apoptotic bodies—bodies that are quickly phagocytosed by macrophages or nearby cells—are its distinguishing features (Ghavami et al., 2014). It is interesting that the Bcl-2 family of proteins and other regulatory elements such as AMP-activated protein kinase (AMPK) that are shared by both apoptosis and autophagy (Pattingre et al., 2005). The variety of cell death routes, which have overlapping and different molecular causes, as well as the limited therapeutic window for some types of neuronal cell death, are barriers to effective therapy against neurotrauma-induced neuronal cell death (Faden, 2002).

At present time, there are no effective therapies available for TBI patients receiving clinical intervention. Oral supplementation with vegetal bioactive compounds shows promise in delaying the irreversible course in this discouraging situation (Stacchiotti and Corsetti, 2020). However, given that the “one-drug, one-target” approach to treating the complex pathophysiology of traumatic brain injury (TBI) has not proven to be effective in clinical settings, traditional medicinal herbs or plants could have a pleiotropic effects and may offer a viable therapeutic supplementation (Di Paolo et al., 2019). Various substances have been employed thus far to control autophagic activity after traumatic brain injury. For instance, apocynin, quercetin, luteolin, polyphenols baicalin and more are found in a wide variety of fruits and vegetables as a modulator of TBI-related neuronal injury (Zeng et al., 2020). The botanical name for frankincense is *Boswellia sacra Fluck*, and it is a member of the Burseraceae family. The majority of these Boswellia species’ chemical components are comparable. The most widely used type of Boswellia in Arab nations is *Boswellia sacra*, often known as “*Omani Luban*” which has long been used to cure a variety of illnesses (Al-Yahya et al., 2020; Alyahya and Asad, 2020). Acetyl-11-keto-beta-boswellic acid (AKBA) and 11-keto-beta-boswellic acid (KBA), which have been investigated for their possible pharmacological and therapeutic qualities, are the two most powerful anti-inflammatory boswellic acids found in Boswellia (Asad and Alhomoud, 2016). The bioactive phytoconstituents of boswellia, boswellic acids and pentacyclic triterpenoids have demonstrated encouraging outcomes in both experimental and clinical research. It is thought to be a potentially useful natural pharmacophoric molecule that could be important for finding anti-inflammatory and therapeutic drugs (Iram et al., 2017). It is traditionally used to cure stomach, skin, ear, and throat infections, to relieve menstruation pain, cardiovascular and neurological issues, etc. It is also chewed as a mouth freshener in many nations. Additionally, goods derived from Boswellia oleo gum resin are sold all over the world for a variety of purposes (Hamidpour et al., 2013; Liu et al., 2018; Mojaverrostami et al., 2018). In this study, we examined the neuroprotective effects of *Boswellia Sacra Resin* (BSR) against apoptosis TBI-induced with a particularly attention to autophagic flux modulation.

## 2 Materials and methods

### 2.1 Reagents and gases

Acetone, acetonitrile, and formic acid (purity > 99.9%) were purchased from Sigma Aldrich (Amsterdam, Holland); hydrochloric acid was purchased from Carlo Erba (Milan, Italy). The standard solutions (purity > 99.9%) at 1,000 mg L−1 of gallic acid, catechin, caffeic acid, syringic acid, rutin, ellagic, hesperidin, ferulic acid, myricetin, quercetin, apigenin, naringenin and kaempferol were purchased from Sigma-Aldrich S. r.l. (Milan, Italy); chlorogenic acid was purchased from VWR (Milan, Italy). Apigenin and kaempferol were dissolved in aqueous solution at pH > 8.

### 2.2 Sample extraction

The sample extraction was carried out according to protocols previously reported (Puigventos et al., 2015). In brief, 0.1 g of sample was weighted and added to 10 mL of acetone/water/hydrochloridric acid solution (70:29:0.1 v/v/v). The mixture was sonicated for 30 min. Subsequently, the mixture was centrifugated for 15 min at 3,500 rpm, and the supernatant filtered with 0.45 μm nylon filters and stored at −4°C until the analysis.

### 2.3 Materials

Oleo gum resins were collected from verified Boswellia sacra Fluck trees of Wadi Doka (Najdi type resin) on the plateau region north of Salalah during 2023. The sample was collected by traditional method. This region experiences a desert climate, with low rainfall (<100 mm annually) and sharp temperature variations throughout the day. The oleo gum resin was authenticated by comparison with preserved voucher samples stored at the Herbarium of Nizwa University, Oman. Unless otherwise stated, all compounds were purchased from Sigma-Aldrich.

### 2.4 LC-HRMS conditions and validation of the method

The chromatographic separations were carried out as reported before with a Raptor C18 column (2.1 mm × 100 mm, 1.7 μm) (Cammilleri et al., 2023). The mobile phase consisted of eluent A: H_2_O + formic acid 1%, eluent B: acetonitrile + formic acid 1% for a total run time of 14 min with a flow rate of 0.3 mL min^-1^.

As a mass spectrometer, a Q ExactiveTM Plus Hybrid Quadrupole-Orbitrap™ (Thermo Fisher Scientific, California, United States) was employed The Full MS scan/dd-MS^2^–SIM mode was used to collect all data. The resolution of the Orbitrap was adjusted to 70,000 FWHM (scan range 100–1,000 m/z). For a maximum injection period of 200 ms, the automatic gain control (AGC) was set to 3 × 10^6^ ions. The product ions were discovered by raising the normalized collision energy until the precursor ions were completely fragmented. Each analyte was assigned a normalized collision energy (NCE) value. The retention time (tR), accurate mass, and distinctive fragmentation were used to identify the analytes. Each day before the study, an external calibration for mass accuracy was done. The Thermo Xcalibur ™ version 4.0 software was used to record and expound on acquisition data. The method’s performance was evaluated for linearity, specificity, and trueness in compliance with Commission Decision 2002/657. The limits of detection and quantification (LODs and LOQs) were determined by the 3σ and 10σ approach. The linearity test yielded good results for all analytes tested (r^2^ > 0.993). Trueness by recovery yielded values ranging between 80% and 105%. The polyphenols concentrations were expressed as µg/Kg.

### 2.5 Extraction of the Boswellia sacra gum resin (BSR) acid fraction

The particle size of the harvested oleo gum of Boswellia sacra resin (BSR) was reduced to a coarse powder with a mortar and a pestle for 2 hours. A fine powder was produced with an electrical grinder. 200 g were placed into a 5,000 mL bottom flask, 2 L of distilled water were added. A hydro distillation with a Clevenger type apparatus was performed under atmospheric pressure. The resulting essential oil was collected (14.2 mL). After 6–8 h no further increase of essential oil was observed. The remaining mixture was filtered (Whatman filter paper, grades 1,2 and 3), the residue was washed out with hot water 3–4 times. The filtrate was cold down to 0°C to obtain a precipitate. After 60 min the precipitate was collected and washed out several times with cold distilled water, dried under vacuo and powdered with the electrical grinder. To reduce the water content below the powder was transferred into a desiccator and this is followed by sieving the powder into a very fine mesh at 40°C for 5 days. The final particle size (3–5 mm) the resulting BSR acid fraction (80 g) was produced by grinding the material at a temperature below 0°C.

### 2.6 HPLC analysis of BSR for pentacyclic triterpenic acids

For chemical characterization of the BSR acid fraction, eight pentacyclic triterpenic acids (PTA), alpha-boswellic acid (alpha-BA), acetyl-alpha-boswellic acid (alpha-ABA), beta-boswellic acid (beta-BA),acetyl-Beta-boswellic acid (B-ABA), 11-keto-beta-boswellic acid (KBA), acetyl-11-keto-beta-boswellic acid (AKBA), lupeolic acid (LA), and acetyl-lupeolic acid (ALA), were quantified by HPLC analysis. For detailed information please see our previous work (Schmiech et al., 2019).

### 2.7 Animals

CD1 male mice (8-week-old, 18–24 g) were acquired from Envigo (Milan, Italy) and located in a controlled environment and provided with standard rodent chow (Teklad standard diet acquire from Envigo) and water available *ad libitum*. They were housed 5 mice/cage and maintained in a 12:12 h light–dark cycle at 21°C ± 1°C and 50% ± 5% humidity. The University of Messina Review Board for animal care (OPBA) approved the study (P.R. 89126.8).

### 2.8 Experimental design and groups

The controlled impactor device Impact OneTM Stereotaxic impactor for controlled cortical impact (CCI) (Leica, Milan, Italy) was used to create a cortical contusion on the exposed cortex after a craniotomy (tip diameter: 4 mm; cortical contusion depth: 3 mm; impact velocity: 1.5 m/s). The clinical symptoms and weight of the animals were monitored daily and recorded. Sham mice underwent the identical surgical procedure but were not injured (Impellizzeri et al., 2017; Fusco et al., 2020; Campolo et al., 2021).

Mice were divided as following:

• Sham + vehicle group: mice were subjected to the surgical procedures as above except that the impact was not applied, and animals were treated o. s. with vehicle (data not shown).
• Sham + BSR: mice were subjected to the surgical procedures as above except that the impact was not applied, and animals were treated o. s. with 500 mg/kg on BSR in saline 1 h after TBI medical procedures.
• TBI: mice were subjected to CCI plus administration of vehicle (saline).
• TBI + BSR: As for the TBI + vehicle group but BSR was administered o. s. at 500 mg/kg in saline 1 h after TBI.

Taking into account that there is no discernible difference between the Sham and Sham + BSR groups we choose to shown in the figures Sham + BSR group. The animals of the first set of experiment were sacrificed 24 h after TBI induction. The animal of the second group pf experiment were sacrificed 30 days after TBI induction, and they received every days for 30 days starting 1 h after the damage orally administration of BSR at the dose of 500 mg/kg (see Supplementary Material for experimental design graph).

### 2.9 Behavioural analysis

30 days after the trauma induction, a designed group of animals underwent behavioural testing. Mice were moved to the behaviour testing room 30 min before the first trial started so they could become accustomed to the environment. Based on behavioural tests that were used to keep the environment as uniform as feasible, animals were trained to use the equipment before every recording. The behavioural tests were conducted by three distinct trustworthy experts who were blinded to the animals’ damage state. Below a brief description of tests.

#### 2.9.1 Force swimming test (FST)

The method is based on that which Porsolt et al. described (Porsolt et al., 1979). Briefly, FST is used to assess depressive-like conditions. Mice are placed in an impenetrable, transparent tank filled with water, and their movement behaviour related to escape is recorded. In this experiment, for 6 minutes, each mouse was gently placed in the cylinder, and the duration of floating was recorded. During the final 4-min of the test, immobility was examined (Genovese et al., 2021).

#### 2.9.2 Open field test (OFT)

The OFT, created by Calvin S. Hall, is an experiment that measures a rodent’s general locomotor activity levels, anxiety, and exploratory willingness. Each mouse in this experiment was trained before being put in the centre of the box, where activity was then recorded for 5 minutes of exploration (Prut and Belzung, 2003).

#### 2.9.3 Elevated plus maze (EPM)

Utilizing the Elevated Plus Maze (EPM) test, rodents’ anxiety-related behaviour is evaluated. The EPM device is made up of a core region, two oppositely positioned open arms, two oppositely positioned closed arms, with an elevated "+"-shaped maze. A video camera set above the maze records the subjects’ actions while they freely navigate it, and their actions are then analysed. After training, it was counted how many times the mice entered each arm and how long they spent in open arms (Pellow et al., 1985).

#### 2.9.4 Morris Water Maze (MWM)

Hippocampal-dependent spatial learning and memory were assessed using the MWM test (Zhao et al., 2017; Siebold et al., 2020). Following a training session, a mouse was placed in the water in each of the three separate quadrants and given 1 minute to swim there. The platform was taken away for the test 1 day following the navigation experiment. It was noted how much time was spent in the target quadrant.

#### 2.9.5 Novel object recognition (NOR)

The NOR test was used to determine whether mice had a natural tendency to spend time studying unfamiliar or familiar objects. Mice were placed in the box for 5 min after a training session, during which the examiner replaced one of the familiar objects with a novel one at random. Each object’s total amount of mouse exploration time was recorded (Siracusa et al., 2017; Pan et al., 2018).

### 2.10 Histological brain analysis

After the experiment, brain tissue was removed, fixed at room temperature in buffered formaldehyde solution (10% in phosphate buffered saline), dehydrated by graduated ethanol, and then embedded in paraffin. Light microscopy was used to examine tissue sections that were 7 um thick after being deparaffinized with xylene and stained with haematoxylin/eosin (Bio-Optica, Milan, Italy). The number of damaged neurons was counted, and the grey matter’s histopathologic alterations were graded on a 6-point scale: No lesion was found, 1; 1–5 eosinophilic neurons were present in the Gray matter, 2; 5–10 eosinophilic neurons were present, 3; more than 10 eosinophilic neurons were present, 4; a small infarction (less than one third of the grey matter area), 5; a moderate infarction (one third to one half of the Gray matter area); and 6, a large infarction (more than half of the grey matter area). To determine a final score for each mouse, the results from every part of each brain were averaged. The slices were then analysed by a blinded histopathologist using an optical microscope using a Leica DM6 microscope (Leica Microsystems Spa, Milan, Italy) (Petrosino et al., 2017).

### 2.11 Cytokines measurement

Using commercially available enzyme-linked immunosorbent assay (ELISA) kits (R&D Systems, Minneapolis, MN, United States) in accordance with the manufacturer’s instructions, TNF-α, IL-1β, and IL-6 levels from brain were measured as previously described (Cordaro et al., 2020a).

### 2.12 Antioxidants and malondialdehyde measurement

The supernatant of the brain tissue homogenate was centrifuged (14,000 rpm at 4°C for 30 min) as previously described (Marklund and Marklund, 1974; Rajasankar et al., 2009). ELISA kits (R&D Systems, Minneapolis, MN, United States) were used to measure superoxide dismutase (SOD) and glutathione (GSH-Px) levels. The test procedure was described in detail in the manufacturer’s manuals. Levels of malondialdehyde in brain tissue were determined as an indicator of lipid peroxidation (Ohkawa et al., 1979). Briefly, brain tissues were weighed and homogenized in a 1.15% (wt/vol) KCl solution. 100 μL aliquots of homogenate were then removed and added to a reaction mixture containing 200 μL 8.1% (wt/vol) lauryl sulfate, 1.5 mL 20% (vol/vol) acetic acid (pH 3.5), 1.5 mL 0.8% (wt/vol) thiobarbituric acid, and 700 μL distilled water. Samples were then boiled for 1 hour at 95°C and centrifuged at 3000g for 10 min. The absorbance of the supernatant was measured spectrophotometrically at 532 nm. MDA levels were expressed as nmol/mg of tissue (Di Paola et al., 2009; Genovese et al., 2022).

### 2.13 Apoptosis and autophagy detection

The level of mRNA expression of apoptosis-related cytokines caspase-3, caspase-8, caspase-9, Bax, Bcl-2, and cytochrome c and autophagy markers such as Beclin-1, LC3 AMPK and p62 were determined using real-time quantitative RT polymerase chain reaction (RT-PCR) as previously described (Liu and Saint, 2002; Hu et al., 2011; Wang et al., 2013; Ze et al., 2014; Wang et al., 2022): Caspase-8 Forward primer ATCTGCTGTATCCCAGC Reverse primer AGG​CAC​TCC​TTT​CTG​GAA​GTT​AC; Caspase-9 Forward primer GCG​GTG​GTG​AGC​AGA​AAG​A Reverse primer CCT​GGG​AAG​GTG​GAG​TAG​GA; Caspase-3 Forward primer CTG​ACT​GGA​AAG​CCG​AAA​CTC Reverse primer GAC​TGG​ATG​AAC​CAC​GAC​CC; Bax Forward primer GGA​TGC​GTC​CAC​CAA​GAA​G Reverse primer CAA​AGT​AGA​AGA​GGG​CAA​CCA​C; Bcl-2 Forward primer TGT​GGT​CCA​TCT​GAC​CCT​CC Reverse primer ACA​TCT​CCC​TGT​TGA​CGC​TCT; Cytochrome c Forward primer CAT​CCC​TTG​ACA​TCG​TGC​TT Reverse primer GGG​TAG​TCT​GAG​TAG​CGT​CGT​G; LC3 Forward primer AAC​GTA​GGC​ACC​CAC​ATA​GG Reverse primer GAA​GAG​ACT​GCC​CCT​GAC​AC; Beclin1 Forward primer GAA​CTC​TGG​AGG​TCT​CGC​T Reverse primer CAC​CCA​GGC​TCG​TTC​TAC​C; p62 Forward primer AGT​CCA​GAA​TTC​CTG​CCT​GA Reverse primer TTC​ATT​CGG​CTT​CAC​ATG​AA; adenosine monophosphate (AMP) activated protein kinase (AMPK) Forward primer GTG​ATC​AGC​ACT​CCG​ACA​GA Reverse primer TCT​CTG​GCT​TCA​GGT​CCC​TA; β-actin Forward primer AAT​GTG​TCC​GTC​GTG​GAT​CTG​A Reverse primer AGT​GTA​GCC​CAA​GAT​GCC​CTT​C.

### 2.14 Western Blots

Cytosolic extracts were prepared as previously described (Cordaro et al., 2017; Di Paola et al., 2021a; Di Paola et al., 2021b). The following primary antibodies were used: anti-Bax (1:500; SCB, B-9 sc-7480), anti-Bcl-2 (1:500; SCB, C-2 sc-7382), Beclin-1 (1:500; SCB, sc-48381) and LC3 (1:500; SCB, sc-271625) in 1× PBS, 5% w/v non-fat dried milk, 0.1% Tween-20 at 4°C overnight (Impellizzeri et al., 2016a; Paterniti et al., 2017; Cordaro et al., 2018; Cordaro et al., 2020b; Crupi et al., 2020). Blots were further probed with an anti-β-actin protein antibody (1:500; SCB) for the cytosolic fraction to make sure that they were loaded with an equivalent number of proteins (Di Paola et al., 2016a; Cordaro et al., 2020c). As directed by the manufacturer, signals were evaluated using an enhanced chemiluminescence (ECL) detection system reagent (Thermo, Monza, Italy) (Akki et al., 2018; Remigante et al., 2022). Using BIORAD ChemiDoc TM XRS + software and densitometry, the relative expression of the protein bands was measured and standardized to the levels of b-actin and lamin A/C (Paterniti et al., 2015; Di Paola et al., 2016b; Esposito et al., 2016; Siracusa et al., 2018; Peritore et al., 2020).

### 2.15 Statistical evaluation

The data in this study are presented as the average ± SEM and represent at least three experiments conducted on various days. N denotes the number of animals utilized in in vivo experiments. The G*Power 3.1 software (Die Heinrich-Heine-Universitat Dusseldorf, Dusseldorf, Germany) was employed to calculate the number of animals used in in vivo research. A competent histopathologist examined the data, without knowledge of the treatments. In all the statistical studies, GraphPad Software Prism 9 (La Jolla, CA, United States) was used. One-way ANOVA was used to examine the data, and then a Bonferroni post-hoc test for multiple comparisons was used. A *p*-value of 0.05 or less was regarded as significant. In figure: ns *p* > 0.05; *p ≤ 0.05; **p ≤ 0.01; ***p ≤ 0.001; ****p ≤ 0.0001.

## 3 Results

### 3.1 Polyphenols contents and HPLC-MS/MS analysis in BSR

**FIGURE 1:**
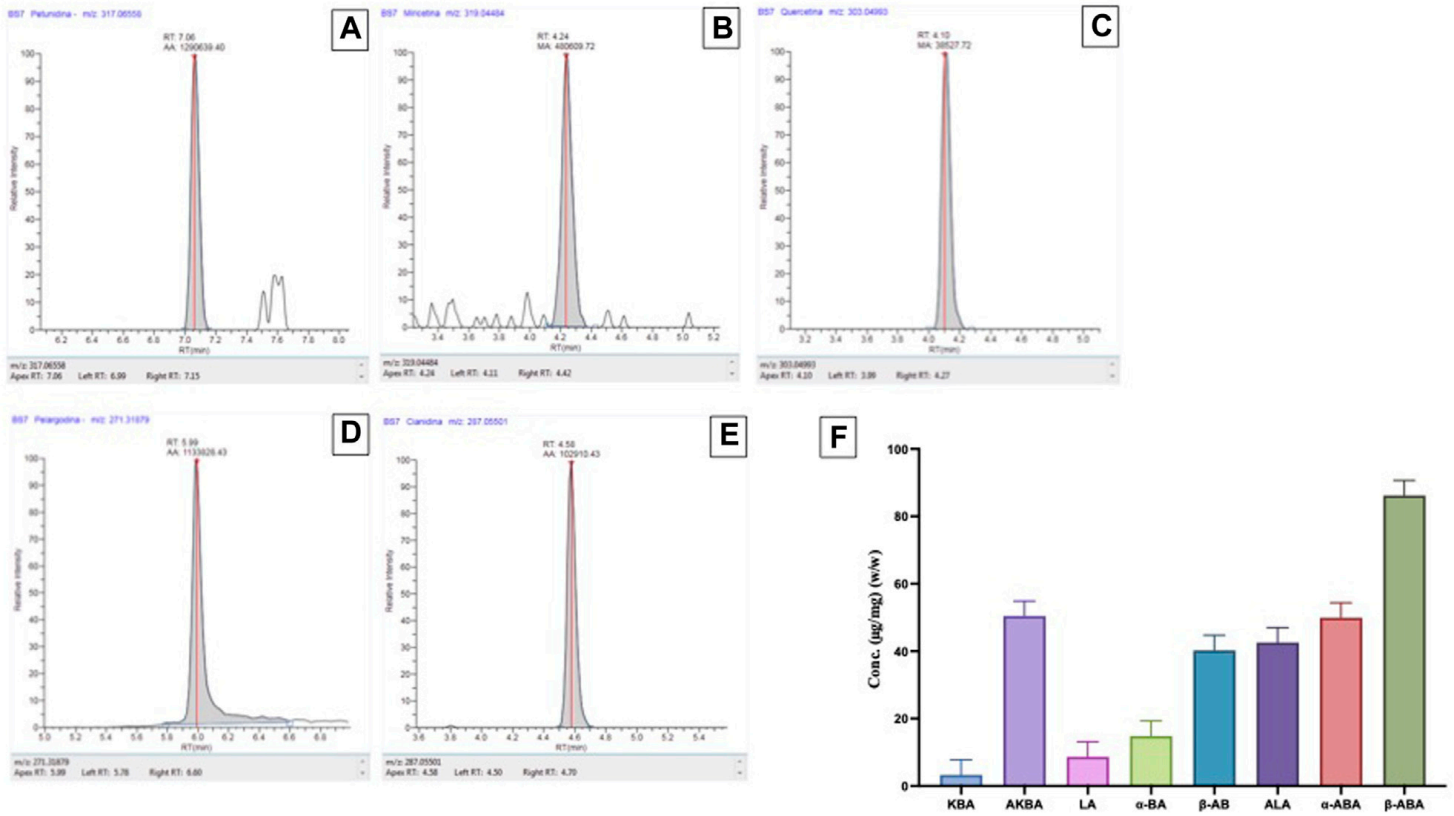
Polyphenols contents found in the BSR. Chromatogram of a B. Sacra sample analyzed by the LC-HRMS method. **(A)** = Petunidin; **(B)** = Myricetin; **(C)** = Quercitin; **(D)** = Pelargodin; **(E)** = Cyanidin. HPLC **(F)** analysis for pentacyclic triterpenic acids: alpha-boswellic acid (alpha-BA), acetyl-alpha-boswellic acid (alpha-ABA), beta-boswellic acid (beta-BA), acetyl-Beta-boswellic acid (B-ABA), 11-keto-beta-boswellic acid (KBA), acetyl-11-keto-beta-boswellic acid (AKBA), lupeolic acid (LA), and acetyl-lupeolic acid (ALA).

The polyphenols contents found in the B. Sacra samples followed the order Petunidin > Pelargonidin > Cyanidin > Myricetin > Quercetin. Among the anthocyanins, a high presence of Petunidin (925.85 μg/Kg) (Figure 1A) was found, followed by Pelargonidin (2.36 μg/Kg) (Figure 1D) and Cyanidin (0.56 μg/Kg) (Figure 1E). Myricetin (47.10 μg/Kg) (Figure 1B) and Quercetin (1.78 μg/Kg) (Figure 1C) were the only flavonols detected. No cinnamate esters, hydroxycinnamic acids and other sub-classes of polyphenols were found. For chemical characterization of the BSR acid fraction were quantified by HPLC analysis eight pentacyclic triterpenic acids: alpha-boswellic acid (alpha-BA), acetyl-alpha-boswellic acid (alpha-ABA), beta-boswellic acid (beta-BA),acetyl-Beta-boswellic acid (B-ABA), 11-keto-beta-boswellic acid (KBA), acetyl-11-keto-beta-boswellic acid (AKBA), lupeolic acid (LA), and acetyl-lupeolic acid (ALA) (Figure 1F).

### 3.2 Effects of BSR on memory performance, locomotor activity changes brought on by TBI, and spatial learning

**FIGURE 2:**
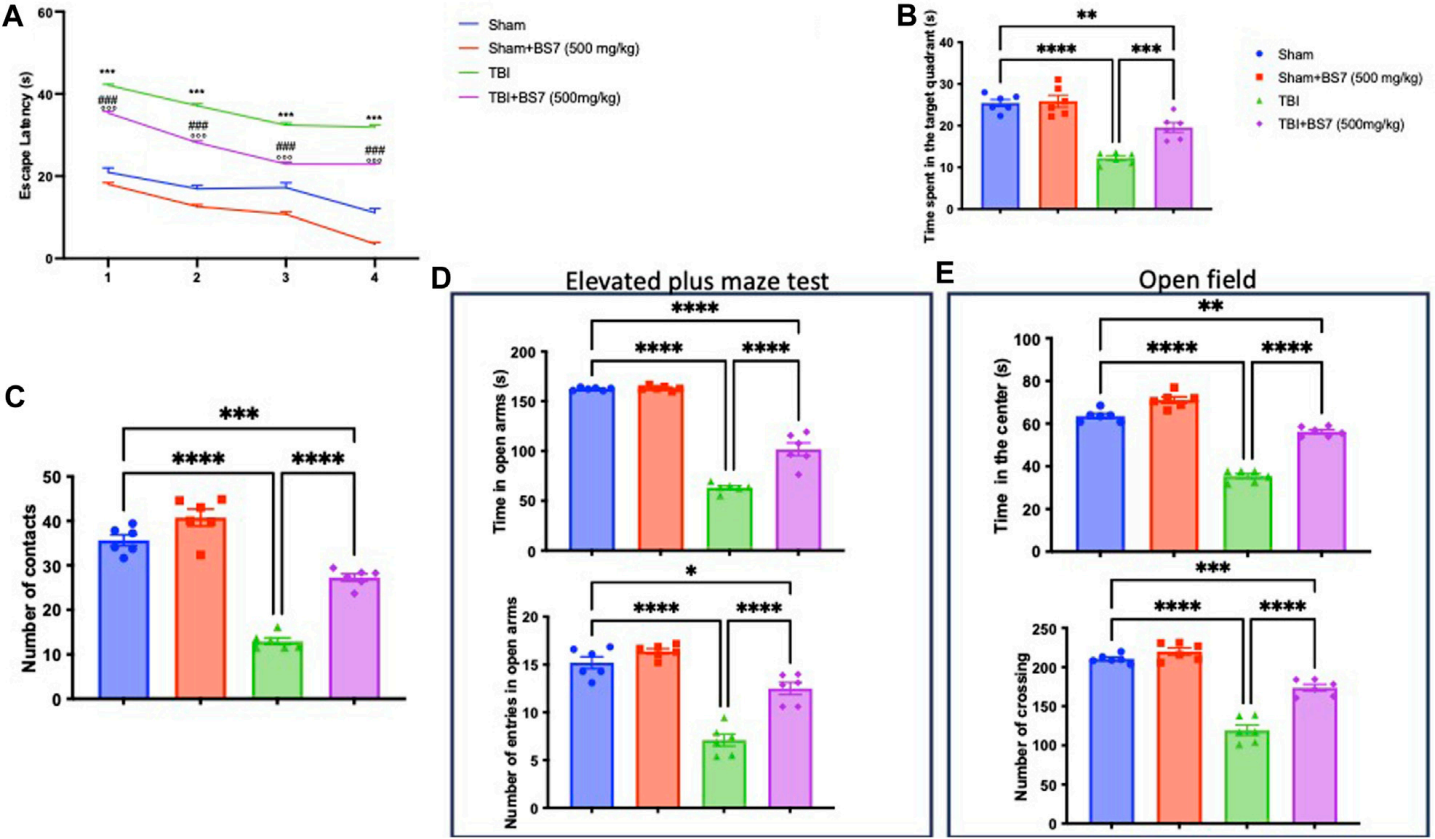
Effects of BSR on spatial learning, memory function, anxiety, and locomotor activity. Morris Water Maze training **(A)** and probe **(B)**; novel object recognition **(C)**; elevated plus maze test **(D)**; Open field **(E)**. As showed in panel 2, BSR administration significantly improve behaviorural recovery in terms of spatial learning, memory function, anxiety, and locomotor activity after TBI. The graphs are representative of at least three experiments performed on different experimental days. Each data is expressed as mean ± S.E.M. from n = 6 male mice for each group. TBI + BS7 vs Sham.

The MWM test was used to determine whether BSR could help with memory problems brought on by TBI. When compared to the controls, TBI-subjected animals took longer to find the platform during training (Figure 2A). In addition, the injured animal spent less time throughout the probe experiment in the target quadrant of the platform (Figure 2B). The escape latency was dramatically decreased (Figure 2A) and the duration spent in the target quadrant was increased (Figure 2B) after oral administration of BSR at a dose of 500 mg/kg, demonstrating an improvement in the cognitive deficiencies brought on by the trauma. We evaluated any shortcomings in their social interaction and exploratory behaviour using the NOR (Figure 2C) test. In this test, we discovered that after TBI, the amount of number of contacts were statistically reduced (Figure 2C). The administration of BSR, on the other hand, considerably improves the memory function harmed by trauma. The EPM test was also applied to mice to evaluate risk-taking behaviours and post-injury anxiety. According to the bibliography, fictitious animals spend more time in open arms whereas injured animals spend more time in closed arms, which also lowers the number of entries. However, compared to the TBI group, the animals that got oral BSR treatment spent longer time in the open arms and made more entrances (Figure 2D). The OFT was utilized to assess locomotor activity further. We found that following TBI injuries, mice spent less time in the centre and made fewer crossings, in contrast to sham animals. In this case, BSR was successful in resuming locomotor activity and the frequency of crossings (Figure 2E).

### 3.3 BSR limits histological alteration induced by TBI

**FIGURE 3:**
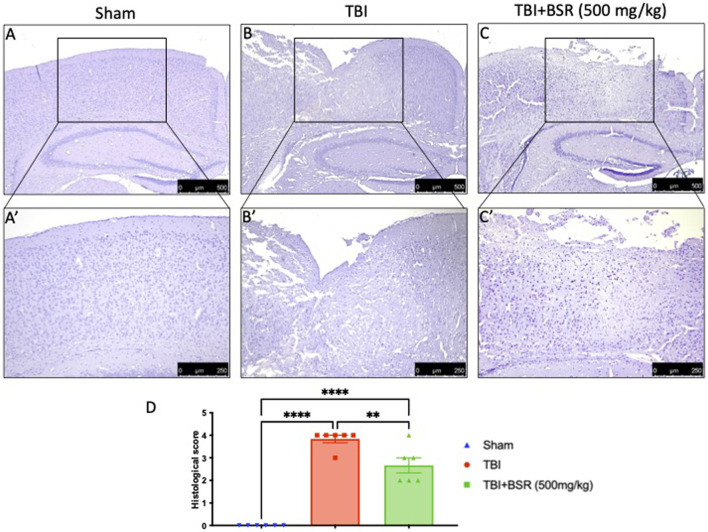
BSR limits histological alteration induced by TBI. Representative images of histological structure of: Sham **(A)** and higher magnification **(A′)** TBI **(B)** and higher magnification **(B′)** and TBI + BSR **(C)** and higher magnification **(C′)**; histological score **(D)**. The figures are representative of at least three experiments performed on different experimental days. Each data is expressed as mean ± S.E.M. from n = 6 male mice for each group.

Histological analysis of a brain sample taken from the TBI group 24 h after the TBI injury revealed significant tissue damage, inflammation, and architectural alterations when compared to brain from the sham group (Figures 3A, A' for sham; Figures 3B, B' for TBI, see histological score 3D). When administered at a dose of 500 mg/kg, BSR significantly lessened the severity of brain injury when compared to the TBI group (Figures 3C, C' see histological score Figure 3D).

### 3.4 BSR administration modulates lipid peroxidation, antioxidant, and cytokines release

**FIGURE 4:**
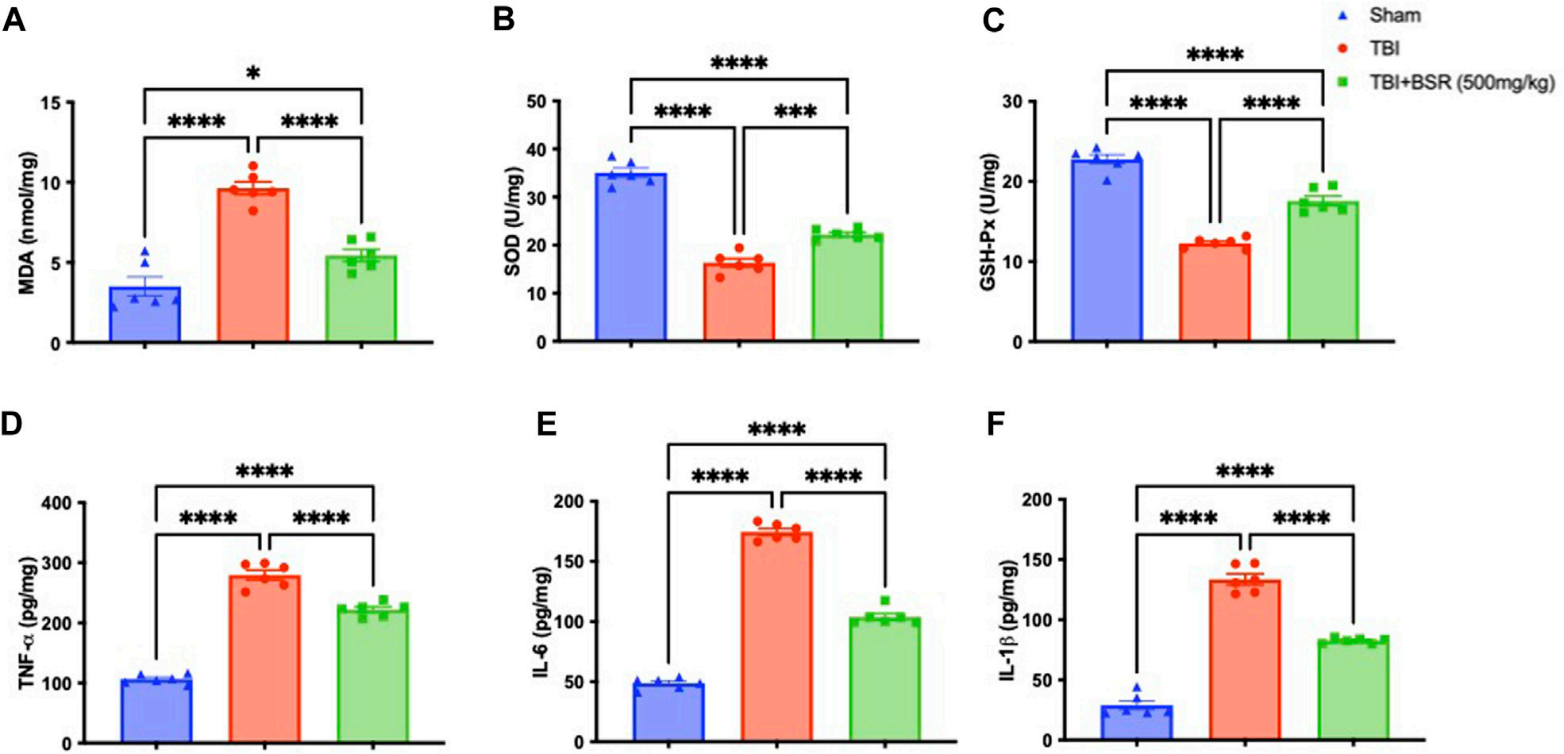
Effects of BSR administration on lipid peroxidation, antioxidant enzymes and cytokines release. MDA **(A)**, SOD **(B)** and GSH-Px activity **(C)** TNF-α **(D)**, IL-6 **(E)**, and IL-1β **(F)**. The graph is representative of at least three experiments performed on different experimental days. Each data is expressed as mean ± S.E.M. from n = 6 male mice for each group.

Given the high concentration of polyunsaturated fatty acids in the brain, lipid peroxidation is the main manifestation of oxidative stress following TBI. Comparing the TBI group to the sham mice, we discovered that there was a considerably higher level of lipid peroxidation that was significantly attenuated following oral administration of BSR (Figure 4A). The cell is shielded from oxidative stress by enzymes that neutralize superoxide and H_2_O_2_. The primary defensive enzymes against superoxide radicals are GSH-Px and SOD (Cordaro et al., 2021a; Cordaro et al., 2021b). Oxidative stress impairs mitochondria’s ability to function and move to synaptic areas, which causes synaptic dysfunction and neurodegeneration. After controlled cortical impact, we observed lower levels of SOD (Figure 4B) and GSH-Px (Figure 4C) compared to sham mice, according to the literature. Following oral administration of BSR at a dose of 500 mg/kg, physiological levels were practically repristinate. Cytokines storm promotes the inflammatory response by activating microglia and increasing the synthesis of chemokines, and preclinical models show that TBI causes neuronal injury with these raised levels (Ahmad et al., 2013; Gugliandolo et al., 2018). We used ELISA kits to measure the levels of TNF-α (Figure 4D), IL-6 (Figure 4E), and IL-1β (Figure 4F). While the sham group had only trace quantities of this cytokine, brain samples from TBI mice had a substantial increase in all cytokines that was significantly reduced after oral administration od BSR at the dose of 500 mg/kg.

### 3.5 BSR limits neuronal death TBI

**FIGURE 5:**
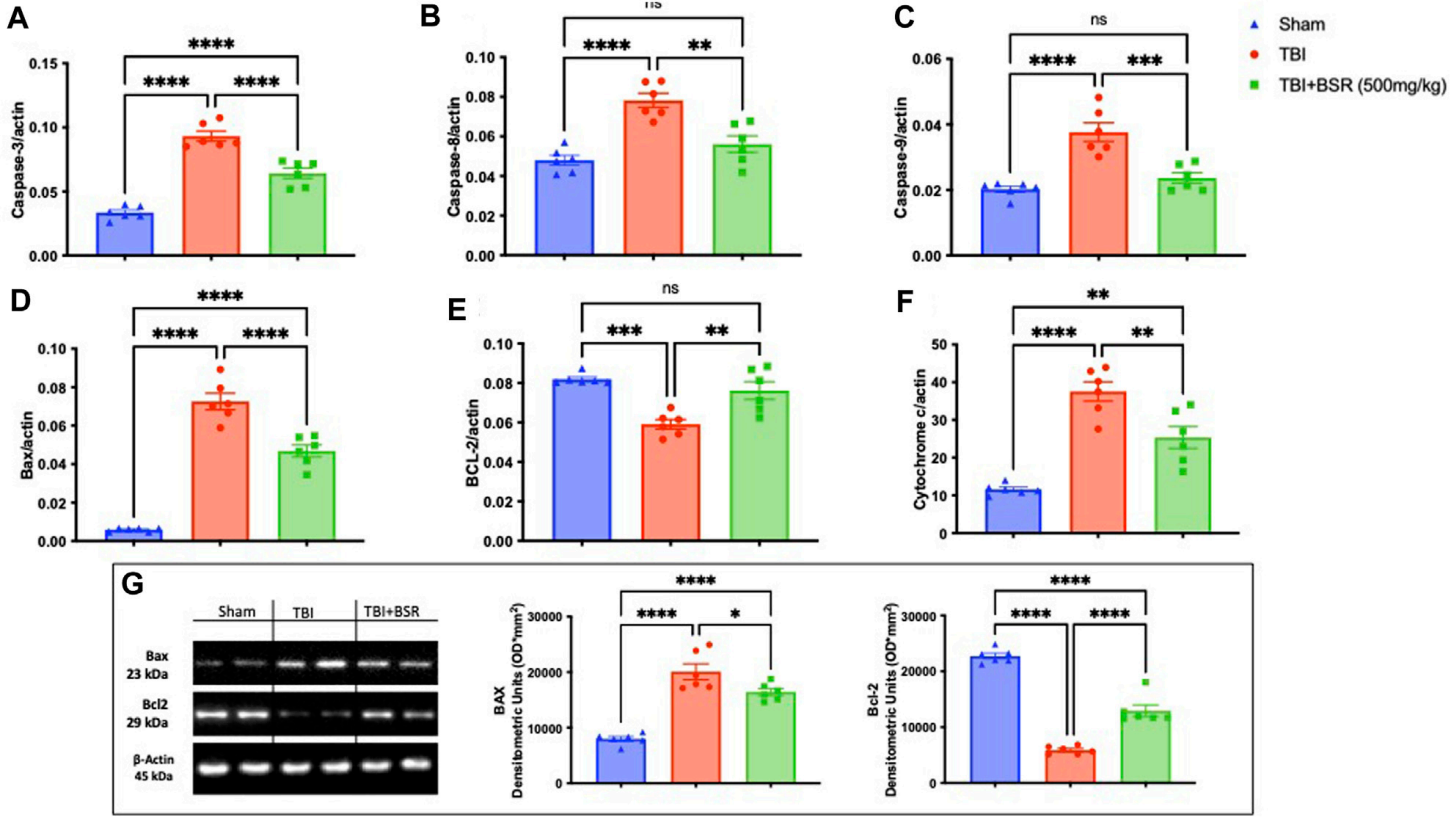
BSR reduced apoptosis TBI-induced. RT-PCR for Caspase-3 **(A)**, Caspase-8 **(B)**, Caspase-9 **(C)**, Bax **(D)**, Bcl-2 **(E)**, and Cytochrome C **(F)**; Western Blots and relative densitometric analysis of Bax and BCL-2**(G)**. The graphs are representative of at least three experiments performed on different experimental days. Each data is expressed as mean ± S.E.M. from n = 6 male mice for each group.

The discovery that caspase-mediated programmed cell death plays a significant role in secondary brain injury raises the possibility of a connection between pathogenic molecular pathways and healing (Jarrahi et al., 2020). For this reason, we made RT-PCR for Caspase-3 (Figure 5A), Caspase-8 (Figure 5B), Caspase-9 (Figure 5C), Bax (Figure 5D), Bcl-2 (Figure 5E), and Cytochrome C (Figure 5F). We found a significantly increase in apoptosis in animal subjected to the injury compared with the sham group except for BCL-2 in which we found a decrease of this expression. The same trend was also observed by western blot analysis of Bax and Bcl-2 (Figure 5G). On the other hand after BSR administration at the dose of 500 mg/kg all the expression of apoptotic marker were brought back to physiological levels.

### 3.6 BSR stimulate autophagic flux

**FIGURE 6:**
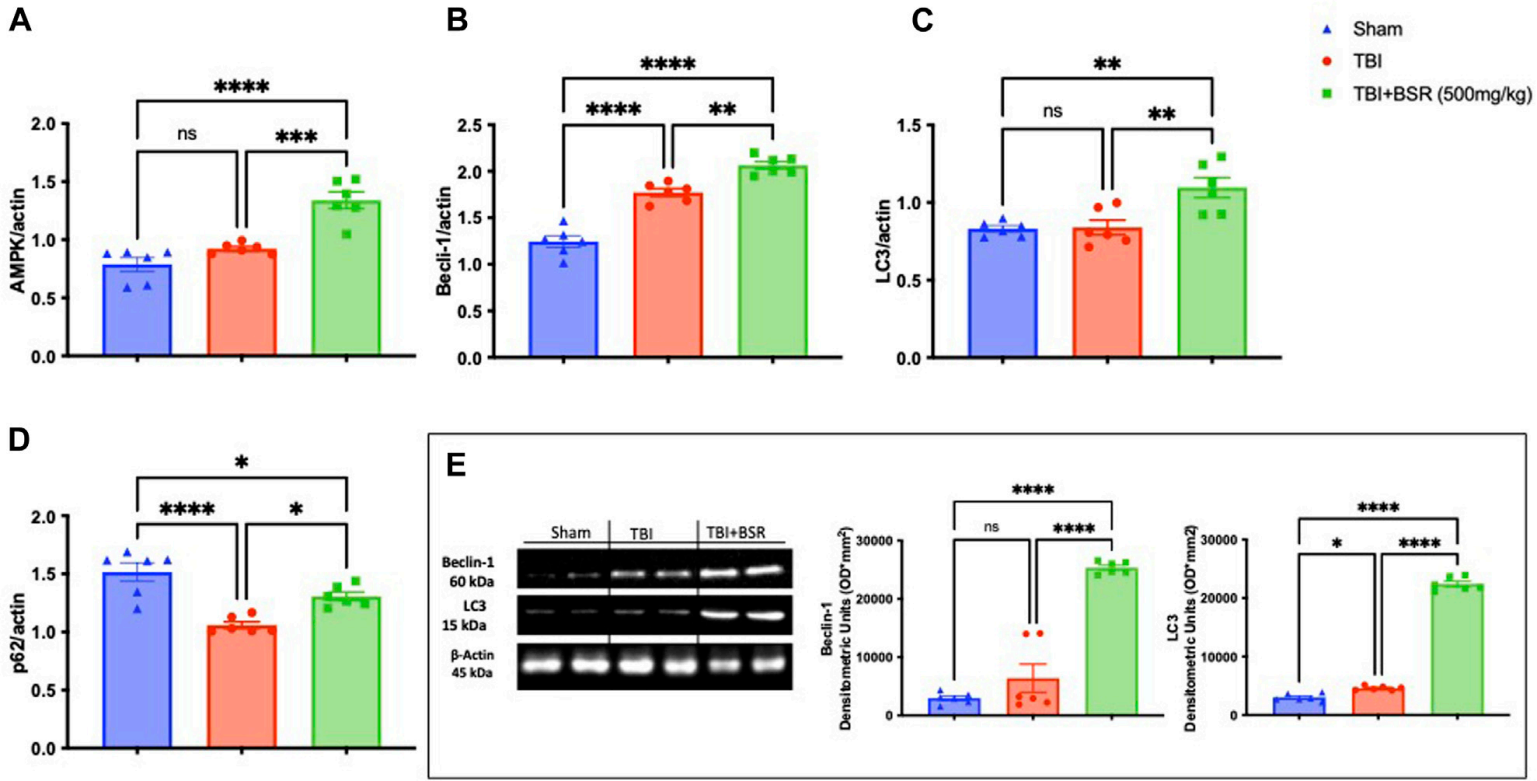
BSR modulates apoptotic and autophagic pathways. RT-PCR for AMPK **(A)**, Beclin-1**(B)**, LC3 **(C)** and p62 **(D)**. Western Blots and relative densitometric analysis of Beclin-1and LC3 **(E)**. The graph is representative of at least three experiments performed on different experimental days. Each data is expressed as mean ± S.E.M. from n = 6 male mice for each group.

Previous study demonstrates that the administration of Boswellia was able to stimulates autophagic flux in an experimental model of rotenone-induced neurotoxicity (Shadfar et al., 2022). In our work we found after RT-PCR analysis that after TBI there were an increase in autophagic flux as demonstrate by AMPK (Figure 6A), Beclin-1 (Figure 6B), LC3 (Figure 6C) and p62 (Figure 6D) compared to the control animals. The same trend was also observed by western blot analysis of Beclin-1 and LC3 (Figure 6E). Additionally, The single oral administration of BSR at the dose of 500 mg/kg significantly increased autophagic flux.

## 4 Discussion

TBI is regarded as a serious health issue that frequently results in mortality and disability and places a significant burden on medical resources. The development of therapeutic methods to treat brain injury was not very rapid. Neuroprotection and neurorecovery are still the primary therapeutic approaches in development, aside from conservative care (Zhang et al., 2014). Studies have shown that secondary cell death, which may eventually make up as much as 40% of the total tissue loss, affects the prognosis after a TBI and so presents a significant pharmacological target for neuroprotective treatment (Smith et al., 2000). Since the dawn of medicine, natural compounds made from plants have been employed in healing. The phytochemicals have undergone substantial evaluation for drug development in recent decades. However, only a small number of these plant species have undergone thorough scientific scrutiny. Therefore, research into the bioactivities of these plants and phytochemicals is necessary. Even now, several of these historically utilized herbs and compounds produced from plants are still useful pharmacologically. One such healing plant is the Burseraceae genus *Boswellia Sacra*. Typically, triterpenoidal principles, essential oils, and carbohydrates make up the normal oleo-gum resin. Boswellic acids include β-boswellic acid, 11-keto-β-boswellic acid, and acetyl-11-keto-β-boswellic acid make up most of the oleo-gum resin. It is safe to use up to oral doses of 1,000 mg/kg in rats, as revealed by Al-Yahya and colleagues, who also showed that the methanolic extract of Boswellia sacra oleo gum resin did not create any significant effect on the kidney and liver with repeated dose administration for 28 days (Al-Yahya et al., 2020). Another study assessed the oral and intraperitoneal toxicity of boswellic acids in mice, rats, and monkeys for acute, subacute, and chronic effects. Boswellic acids were discovered to be safe up to the 2.0 g/kg investigated dosing levels (Khan et al., 2016). With this background in our mind we want to elucidate the molecular pathways by which BSR could have a neuroprotective effects in an experimental model of TBI. It is common practice to examine the prevalent clinical problem in people using animal models of trauma. After a controlled cortical impact, animals that lead to neurological diseases such seizures and deteriorated memory and learning. In our investigation, a single oral dose of BSR at the dose of 500 mg/kg given 1 hour after trauma induction was able to reduce post-traumatic stress disorder symptoms such anxiety and altered locomotor activity while also improving spatial learning and memory. CCI is a consolidated models of brain trauma that induce a significantly alteration in histological architecture (Campolo et al., 2014; Impellizzeri et al., 2016b; Cordaro et al., 2016; Impellizzeri et al., 2017; Gugliandolo et al., 2018; Fusco et al., 2020; Cordaro et al., 2021a; Cordaro et al., 2021b). In our study we found that in the mice subjected to the trauma the perilesional area revealed considerable tissue damage, inflammation, and architecture alterations 24 h after TBI injury that was significantly reduced after the administration of BSR at the dose of 500 mg/kg. A common underlying cause of many neuropathologies is the overproduction of reactive oxygen species (ROS), reactive nitrogen species (RNS), and cytokines which have been demonstrated to harm a variety of cellular components, including proteins, lipids, and DNA. Superoxide dismutase (SOD) and reduced glutathione (GSH), two endogenous defensive enzyme systems, can be overwhelmed by free radicals, especially superoxide (O^2-^), and non-radicals such hydrogen peroxide (H_2_O_2_) (Slemmer et al., 2008). In our study we found a significantly increase in lipid peroxidation as well as in pro inflammatory cytokines in animals subjected to the injury compared to the control group and a significantly reduction in physiological antioxidant system as demonstrated by the analysis of SOD and GSH-Px. On the other hands, a single oral administration of BSR, have been significantly limited these alterations. The three main types of cell death are necrosis, apoptosis, and autophagy. Apoptosis, in contrast to necrosis, is a tightly controlled and energy-intensive process that can be started by the original necrosis. We concentrated on apoptosis and autophagy because there were no specific ways to identify necrosis. The pathophysiology of brain injury in the TBI model heavily depends on apoptosis. The relative amounts of these genes, Bcl-2 and Caspases, which are commonly regarded as the most significant apoptotic regulators, influence the fate of cells (Zhang et al., 2014). In our study we found a significantly increase in apoptotic pathway as demonstrated by the increase in Caspase-3, Caspase-8, Caspase-9, Bcl-2 and Cytochrome C (and obviously in a decrease of Bcl-2) founded in mice subjected to the trauma compared to the control group. After the single administration of BSR we found an important return to the physiological levels of the apoptotic pathway. Numerous and various experimental models of brain injury, including trauma, show increased autophagy (Wang et al., 2013). It is unknown, though, whether autophagy plays a beneficial or harmful function in the recovery of brain-damaged neuronal tissue (Raghupathi, 2004). It is likely that the function of autophagy following brain damage depends on the cell’s ability to react to the accumulation of broken or dysfunctional macromolecules and organelles. Enhancing autophagy would probably be advantageous if the increase in autophagic capacity is minimal (Zhang et al., 2005). Although maintaining ATP homeostasis and controlling metabolism are two of AMPK’s most well-known jobs, it has recently been suggested that AMPK also controls cell apoptosis or survival under stressful circumstances. Independently of the stimuli, AMPK activation can induce the autophagic process (Villanueva-Paz et al., 2016). Moreover, its well know that the increasing of microtubule-associated protein light chain 3 (LC3)-III and beclin-1, while a decreasing in p62 are autophagy markers demonstrating that autophagic activity is persistently activated after TBI in a controlled cortical impact (CCI) system model of TBI *in vivo* and *in vitro* (Liu et al., 2008; Au et al., 2017; Sebastiani et al., 2017). In our study we found a physiological activation of autophagic flux that were significantly improved after BSR administration as demonstrated by the analysis of AMPK, Beclin-1 and LC3. Additionally, cytoplasmic organoids are ubiquitinated by the adaptor protein p62 before being transported to the autophagosome and destroyed by the autolysosome. As a result, the downregulation of p62 points to an autophagic flux (Klionsky et al., 2016). According to bibliography, in our work we found a decrease in p62 in the animals subjected to the trauma compared to the control group that were significantly restored after BSR administration at the dose of 500 mg/kg.

## 5 Conclusion

Acute neuroprotective treatments try to stop the molecular chain reaction that results in damage after TBI. Although neuroprotection is a key strategy for treating this injury, no efficient neuroprotective medications have been discovered from TBI clinical trials to date. However, additional research is required to fully understand the cascade of events that starts with the impact and continues throughout the patient’s life. Using natural substances is the only way to completely avoid all the negative effects of pharmacological therapy. Future directions of our research could include testing BSR on many components of trauma that have not yet been considered to see if it can function on several fronts due to the special combination of this molecule.

## Data Availability

The original contributions presented in the study are included in the article/Supplementary Material, further inquiries can be directed to the corresponding author.

## References

[B1] Ahmad A. Crupi R. Campolo M. Genovese T. Esposito E. Cuzzocrea S. (2013). Absence of TLR4 reduces neurovascular unit and secondary inflammatory process after traumatic brain injury in mice. PLoS One 8 (3), e57208. 10.1371/journal.pone.0057208 23555560 PMC3610903

[B2] Akki R. Siracusa R. Morabito R. Remigante A. Campolo M. Errami M. (2018). Neuronal-like differentiated SH-SY5Y cells adaptation to a mild and transient H(2) O(2) -induced oxidative stress. Cell. Biochem. Funct. 36 (2), 56–64. 10.1002/cbf.3317 29431194

[B3] Alyahya A. A. I. Asad M. (2020). Repeated 28-DAY oral dose study on Boswellia sacra oleo gum resin extract for testicular toxicity in rats. J. Ethnopharmacol. 258, 112890. 10.1016/j.jep.2020.112890 32330512

[B4] Al-Yahya A. A. I. Asad M. Sadaby A. Alhussaini M. S. (2020). Repeat oral dose safety study of standardized methanolic extract of Boswellia sacra oleo gum resin in rats. Saudi J. Biol. Sci. 27 (1), 117–123. 10.1016/j.sjbs.2019.05.010 31889825 PMC6933286

[B5] Asad M. Alhomoud M. (2016). Proulcerogenic effect of water extract of Boswellia sacra oleo gum resin in rats. Pharm. Biol. 54 (2), 225–230. 10.3109/13880209.2015.1028553 25853959

[B6] Au A. K. Aneja R. K. Bayır H. Bell M. J. Janesko-Feldman K. Kochanek P. M. (2017). Autophagy biomarkers beclin 1 and p62 are increased in cerebrospinal fluid after traumatic brain injury. Neurocrit Care 26 (3), 348–355. 10.1007/s12028-016-0351-x 28000126

[B7] Bramlett H. M. Dietrich W. D. (2007). Progressive damage after brain and spinal cord injury: pathomechanisms and treatment strategies. Prog. Brain Res. 161, 125–141. 10.1016/S0079-6123(06)61009-1 17618974

[B8] Cammilleri G. Calabrese V. Pantano L. Brunone M. Galluzzo F. G. Pulvirenti A. (2023). Polyphenols of white lupin (Lupinus albus L.) seeds cultivated in Southern Italy by a LC-HRMS method. Nat. Prod. Res., 1–5. 10.1080/14786419.2023.2245535 37674402

[B9] Campolo M. Crupi R. Cordaro M. Cardali S. M. Ardizzone A. Casili G. (2021). Co-ultra PEALut enhances endogenous repair response following moderate traumatic brain injury. Int. J. Mol. Sci. 22 (16), 8717. 10.3390/ijms22168717 34445417 PMC8395716

[B10] Campolo M. Esposito E. Ahmad A. Di Paola R. Paterniti I. Cordaro M. (2014). Hydrogen sulfide-releasing cyclooxygenase inhibitor ATB-346 enhances motor function and reduces cortical lesion volume following traumatic brain injury in mice. J. Neuroinflammation 11, 196. 10.1186/s12974-014-0196-1 25472548 PMC4265354

[B11] Cordaro M. Cuzzocrea S. Crupi R. (2020c). An update of palmitoylethanolamide and luteolin effects in preclinical and clinical studies of neuroinflammatory events. Antioxidants (Basel) 9 (3), 216. 10.3390/antiox9030216 32150935 PMC7139331

[B12] Cordaro M. D'Amico R. Morabito R. Fusco R. Siracusa R. Peritore A. F. (2021b). Physiological and biochemical changes in NRF2 pathway in aged animals subjected to brain injury. Cell. Physiol. Biochem. 55 (2), 160–179. 10.33594/000000353 33811485

[B13] Cordaro M. Fusco R. D'Amico R. Siracusa R. Peritore A. F. Gugliandolo E. (2020b). Cashew (anacardium occidentale L.) nuts modulate the Nrf2 and NLRP3 pathways in pancreas and lung after induction of acute pancreatitis by cerulein. Antioxidants (Basel) 9 (10), 992. 10.3390/antiox9100992 33066525 PMC7602264

[B14] Cordaro M. Impellizzeri D. Paterniti I. Bruschetta G. Siracusa R. De Stefano D. (2016). Neuroprotective effects of Co-UltraPEALut on secondary inflammatory process and autophagy involved in traumatic brain injury. J. Neurotrauma 33 (1), 132–146. 10.1089/neu.2014.3460 25046306

[B15] Cordaro M. Paterniti I. Siracusa R. Impellizzeri D. Esposito E. Cuzzocrea S. (2017). KU0063794, a dual mTORC1 and mTORC2 inhibitor, reduces neural tissue damage and locomotor impairment after spinal cord injury in mice. Mol. Neurobiol. 54 (4), 2415–2427. 10.1007/s12035-016-9827-0 26960330

[B16] Cordaro M. Scuto M. Siracusa R. D'amico R. Filippo Peritore A. Gugliandolo E. (2020a). Effect of N-palmitoylethanolamine-oxazoline on comorbid neuropsychiatric disturbance associated with inflammatory bowel disease. FASEB J. 34 (3), 4085–4106. 10.1096/fj.201901584RR 31950563

[B17] Cordaro M. Siracusa R. Crupi R. Impellizzeri D. Peritore A. F. D'Amico R. (2018). 2-Pentadecyl-2-Oxazoline reduces neuroinflammatory environment in the MPTP model of Parkinson disease. Mol. Neurobiol. 55 (12), 9251–9266. 10.1007/s12035-018-1064-2 29656363

[B18] Cordaro M. Trovato Salinaro A. Siracusa R. D'Amico R. Impellizzeri D. Scuto M. (2021a). Hidrox^®^ roles in neuroprotection: biochemical links between traumatic brain injury and alzheimer's disease. Antioxidants (Basel) 10 (5), 818. 10.3390/antiox10050818 34065584 PMC8161307

[B19] Crupi R. Cordaro M. Cuzzocrea S. Impellizzeri D. (2020). Management of traumatic brain injury: from present to future. Antioxidants (Basel) 9 (4), 297. 10.3390/antiox9040297 32252390 PMC7222188

[B20] Di Paola D. Capparucci F. Lanteri G. Cordaro M. Crupi R. Siracusa R. (2021b). Combined toxicity of xenobiotics bisphenol A and heavy metals on zebrafish embryos (*Danio rerio*). Toxics 9 (12), 344. 10.3390/toxics9120344 34941778 PMC8706782

[B21] Di Paola D. Iaria C. Capparucci F. Cordaro M. Crupi R. Siracusa R. (2021a). Aflatoxin B1 toxicity in zebrafish larva (*Danio rerio*): protective role of hericium erinaceus. Toxins (Basel) 13 (10), 710. 10.3390/toxins13100710 34679002 PMC8541241

[B22] Di Paola R. Cordaro M. Crupi R. Siracusa R. Campolo M. Bruschetta G. (2016b). Protective effects of ultramicronized palmitoylethanolamide (PEA-um) in myocardial ischaemia and reperfusion injury *in vivo* . Shock 46 (2), 202–213. 10.1097/SHK.0000000000000578 26844976

[B23] Di Paola R. Crisafulli C. Mazzon E. Genovese T. Paterniti I. Bramanti P. (2009). Effect of PD98059, a selective MAPK3/MAPK1 inhibitor, on acute lung injury in mice. Int. J. Immunopathol. Pharmacol. 22 (4), 937–950. 10.1177/039463200902200409 20074457

[B24] Di Paola R. Impellizzeri D. Fusco R. Cordaro M. Siracusa R. Crupi R. (2016a). Ultramicronized palmitoylethanolamide (PEA-um(®)) in the treatment of idiopathic pulmonary fibrosis. Pharmacol. Res. 111, 405–412. 10.1016/j.phrs.2016.07.010 27402190

[B25] Di Paolo M. Papi L. Gori F. Turillazzi E. (2019). Natural products in neurodegenerative diseases: a great promise but an ethical challenge. Int. J. Mol. Sci. 20 (20), 5170. 10.3390/ijms20205170 31635296 PMC6834164

[B26] Esposito E. G B. (2016). A new co-micronized composite containing palmitoylethanolamide and polydatin shows superior oral efficacy compared to their association in a rat paw model of carrageenan-induced inflammation. Eur. J. Pharmacol. 782, 107–118. 10.1016/j.ejphar.2016.03.033 27095683

[B27] Faden A. I. (2002). Neuroprotection and traumatic brain injury: theoretical option or realistic proposition. Curr. Opin. Neurol. 15 (6), 707–712. 10.1097/01.wco.0000044767.39452.bf 12447109

[B28] Fusco R. Gugliandolo E. Siracusa R. Scuto M. Cordaro M. D'Amico R. (2020). Formyl peptide receptor 1 signaling in acute inflammation and neural differentiation induced by traumatic brain injury. Biol. (Basel) 9 (9), 238. 10.3390/biology9090238 PMC7563302 32825368

[B29] Genovese T. Impellizzeri D. D'Amico R. Fusco R. Peritore A. F. Di Paola D. (2022). Role of bevacizumab on vascular endothelial growth factor in apolipoprotein E deficient mice after traumatic brain injury. Int. J. Mol. Sci. 23 (8), 4162. 10.3390/ijms23084162 35456980 PMC9024601

[B30] Genovese T. Siracusa R. Fusco R. D'Amico R. Impellizzeri D. Peritore A. F. (2021). Atrazine inhalation causes neuroinflammation, apoptosis and accelerating brain aging. Int. J. Mol. Sci. 22 (15), 7938. 10.3390/ijms22157938 34360708 PMC8347547

[B31] Ghavami S. Shojaei S. Yeganeh B. Ande S. R. Jangamreddy J. R. Mehrpour M. (2014). Autophagy and apoptosis dysfunction in neurodegenerative disorders. Prog. Neurobiol. 112, 24–49. 10.1016/j.pneurobio.2013.10.004 24211851

[B32] Gugliandolo E. D'Amico R. Cordaro M. Fusco R. Siracusa R. Crupi R. (2018). Neuroprotective effect of artesunate in experimental model of traumatic brain injury. Front. Neurol. 9, 590. 10.3389/fneur.2018.00590 30108544 PMC6079305

[B33] Hamidpour R. Hamidpour S. Hamidpour M. Shahlari M. (2013). Frankincense (rǔ xiāng; boswellia species): from the selection of traditional applications to the novel phytotherapy for the prevention and treatment of serious diseases. J. Tradit. Complement. Med. 3 (4), 221–226. 10.4103/2225-4110.119723 24716181 PMC3924999

[B34] Hu R. Zheng L. Zhang T. Gao G. Cui Y. Cheng Z. (2011). Molecular mechanism of hippocampal apoptosis of mice following exposure to titanium dioxide nanoparticles. J. Hazard Mater 191 (1-3), 32–40. 10.1016/j.jhazmat.2011.04.027 21570177

[B35] Impellizzeri D. Campolo M. Bruschetta G. Crupi R. Cordaro M. Paterniti I. (2016b). Traumatic brain injury leads to development of Parkinson's disease related pathology in mice. Front. Neurosci. 10, 458. 10.3389/fnins.2016.00458 27790086 PMC5061819

[B36] Impellizzeri D. Cordaro M. Bruschetta G. Crupi R. Pascali J. Alfonsi D. (2016a). 2-pentadecyl-2-oxazoline: identification in coffee, synthesis and activity in a rat model of carrageenan-induced hindpaw inflammation. Pharmacol. Res. 108, 23–30. 10.1016/j.phrs.2016.04.007 27083308

[B37] Impellizzeri D. Cordaro M. Bruschetta G. Siracusa R. Crupi R. Esposito E. (2017). N-Palmitoylethanolamine-Oxazoline as a new therapeutic strategy to control neuroinflammation: neuroprotective effects in experimental models of spinal cord and brain injury. J. Neurotrauma 34 (18), 2609–2623. 10.1089/neu.2016.4808 28095731

[B38] Iram F. Khan S. A. Husain A. (2017). Phytochemistry and potential therapeutic actions of Boswellic acids: a mini-review. Asian Pac. J. Trop. Biomed. 7 (6), 513–523. 10.1016/j.apjtb.2017.05.001

[B39] Jarrahi A. Braun M. Ahluwalia M. Gupta R. V. Wilson M. Munie S. (2020). Revisiting traumatic brain injury: from molecular mechanisms to therapeutic interventions. Biomedicines 8 (10), 389. 10.3390/biomedicines8100389 33003373 PMC7601301

[B40] Khan M. A. Ali R. Parveen R. Najmi A. K. Ahmad S. (2016). Pharmacological evidences for cytotoxic and antitumor properties of Boswellic acids from Boswellia serrata. J. Ethnopharmacol. 191, 315–323. 10.1016/j.jep.2016.06.053 27346540

[B41] Klionsky D. J. Abdelmohsen K. Abe A. Abedin M. J. Abeliovich H. Acevedo Arozena A. (2016). Guidelines for the use and interpretation of assays for monitoring autophagy (3rd edition). Autophagy 12, 1–222. 10.1080/15548627.2015.1100356 26799652 PMC4835977

[B42] Liu C. L. Chen S. Dietrich D. Hu B. R. (2008). Changes in autophagy after traumatic brain injury. J. Cereb. Blood Flow. Metab. 28 (4), 674–683. 10.1038/sj.jcbfm.9600587 18059433 PMC2672103

[B43] Liu W. Saint D. A. (2002). Validation of a quantitative method for real time PCR kinetics. Biochem. Biophys. Res. Commun. 294 (2), 347–353. 10.1016/S0006-291X(02)00478-3 12051718

[B44] Liu X. Machado G. C. Eyles J. P. Ravi V. Hunter D. J. (2018). Dietary supplements for treating osteoarthritis: a systematic review and meta-analysis. Br. J. Sports Med. 52 (3), 167–175. 10.1136/bjsports-2016-097333 29018060

[B45] Marklund S. Marklund G. (1974). Involvement of the superoxide anion radical in the autoxidation of pyrogallol and a convenient assay for superoxide dismutase. Eur. J. Biochem. 47 (3), 469–474. 10.1111/j.1432-1033.1974.tb03714.x 4215654

[B46] Mojaverrostami S. Bojnordi M. N. Ghasemi-Kasman M. Ebrahimzadeh M. A. Hamidabadi H. G. (2018). A review of herbal therapy in multiple sclerosis. Adv. Pharm. Bull. 8 (4), 575–590. 10.15171/apb.2018.066 30607330 PMC6311642

[B47] Ohkawa H. Ohishi N. Yagi K. (1979). Assay for lipid peroxides in animal tissues by thiobarbituric acid reaction. Anal. Biochem. 95 (2), 351–358. 10.1016/0003-2697(79)90738-3 36810

[B48] Pan Z. Cui M. Dai G. Yuan T. Li Y. Ji T. (2018). Protective effect of anthocyanin on neurovascular unit in cerebral ischemia/reperfusion injury in rats. Front. Neurosci. 12, 947. 10.3389/fnins.2018.00947 30618576 PMC6297832

[B49] Paterniti I. Campolo M. Siracusa R. Cordaro M. Di Paola R. Calabrese V. (2017). Liver X receptors activation, through TO901317 binding, reduces neuroinflammation in Parkinson's disease. PLoS One 12 (4), e01. 10.1371/journal.pone.0174470 PMC5378346 28369131

[B50] Paterniti I. Di Paola R. Campolo M. Siracusa R. Cordaro M. Bruschetta G. (2015). Palmitoylethanolamide treatment reduces retinal inflammation in streptozotocin-induced diabetic rats. Eur. J. Pharmacol. 769, 313–323. 10.1016/j.ejphar.2015.11.035 26607470

[B51] Pattingre S. Tassa A. Qu X. Garuti R. Liang X. H. Mizushima N. (2005). Bcl-2 antiapoptotic proteins inhibit Beclin 1-dependent autophagy. Cell. 122 (6), 927–939. 10.1016/j.cell.2005.07.002 16179260

[B52] Pellow S. Chopin P. File S. E. Briley M. (1985). Validation of open:closed arm entries in an elevated plus-maze as a measure of anxiety in the rat. J. Neurosci. Methods 14 (3), 149–167. 10.1016/0165-0270(85)90031-7 2864480

[B53] Peritore A. F. Crupi R. Scuto M. Gugliandolo E. Siracusa R. Impellizzeri D. (2020). The role of annexin A1 and formyl peptide receptor 2/3 signaling in chronic corticosterone-induced depression-like behaviors and impairment in hippocampal-dependent memory. CNS Neurol. Disord. Drug Targets 19 (1), 27–43. 10.2174/1871527319666200107094732 31914916

[B54] Petrosino S. Campolo M. Impellizzeri D. Paterniti I. Allarà M. Gugliandolo E. (2017). 2-Pentadecyl-2-Oxazoline, the oxazoline of pea, modulates carrageenan-induced acute inflammation. Front. Pharmacol. 8, 308. 10.3389/fphar.2017.00308 28611664 PMC5448350

[B55] Porsolt R. D. Bertin A. Blavet N. Deniel M. Jalfre M. (1979). Immobility induced by forced swimming in rats: effects of agents which modify central catecholamine and serotonin activity. Eur. J. Pharmacol. 57 (2-3), 201–210. 10.1016/0014-2999(79)90366-2 488159

[B56] Prut L. Belzung C. (2003). The open field as a paradigm to measure the effects of drugs on anxiety-like behaviors: a review. Eur. J. Pharmacol. 463 (1-3), 3–33. 10.1016/s0014-2999(03)01272-x 12600700

[B57] Puigventos L. Navarro M. Alechaga É. Núñez O. Saurina J. Hernández-Cassou S. (2015). Determination of polyphenolic profiles by liquid chromatography-electrospray-tandem mass spectrometry for the authentication of fruit extracts. Anal. Bioanal. Chem. 407 (2), 597–608. 10.1007/s00216-014-8298-2 25370163

[B58] Raghupathi R. (2004). Cell death mechanisms following traumatic brain injury. Brain Pathol. 14 (2), 215–222. 10.1111/j.1750-3639.2004.tb00056.x 15193035 PMC8096005

[B59] Rajasankar S. Manivasagam T. Surendran S. (2009). Ashwagandha leaf extract: a potential agent in treating oxidative damage and physiological abnormalities seen in a mouse model of Parkinson's disease. Neurosci. Lett. 454 (1), 11–15. 10.1016/j.neulet.2009.02.044 19429045

[B60] Ramlackhansingh A. F. Brooks D. J. Greenwood R. J. Bose S. K. Turkheimer F. E. Kinnunen K. M. (2011). Inflammation after trauma: microglial activation and traumatic brain injury. Ann. Neurol. 70 (3), 374–383. 10.1002/ana.22455 21710619

[B61] Remigante A. Spinelli S. Straface E. Gambardella L. Caruso D. Falliti G. (2022). Açaì (*Euterpe oleracea*) extract protects human erythrocytes from age-related oxidative stress. Cells 11 (15), 2391. 10.3390/cells11152391 35954235 PMC9368007

[B62] Schmiech M. Lang S. J. Werner K. Rashan L. J. Syrovets T. Simmet T. (2019). Comparative analysis of pentacyclic triterpenic acid compositions in oleogum resins of different boswellia species and their *in vitro* cytotoxicity against treatment-resistant human breast cancer cells. Molecules 24 (11), 2153. 10.3390/molecules24112153 31181656 PMC6600171

[B63] Schoch K. M. Madathil S. K. Saatman K. E. (2012). Genetic manipulation of cell death and neuroplasticity pathways in traumatic brain injury. Neurotherapeutics 9 (2), 323–337. 10.1007/s13311-012-0107-z 22362424 PMC3337028

[B64] Sebastiani A. Gölz C. Sebastiani P. G. Bobkiewicz W. Behl C. Mittmann T. (2017). Sequestosome 1 deficiency delays, but does not prevent brain damage formation following acute brain injury in adult mice. Front. Neurosci. 11, 678. 10.3389/fnins.2017.00678 29311767 PMC5742218

[B65] Shadfar S. Khanal S. Bohara G. Kim G. Sadigh-Eteghad S. Ghavami S. (2022). Methanolic extract of boswellia serrata gum protects the nigral dopaminergic neurons from rotenone-induced neurotoxicity. Mol. Neurobiol. 59 (9), 5874–5890. 10.1007/s12035-022-02943-y 35804280 PMC9395310

[B66] Siebold L. Krueger A. C. Abdala J. A. Figueroa J. D. Bartnik-Olson B. Holshouser B. (2020). Cosyntropin attenuates neuroinflammation in a mouse model of traumatic brain injury. Front. Mol. Neurosci. 13, 109. 10.3389/fnmol.2020.00109 32670020 PMC7332854

[B67] Siracusa R. Impellizzeri D. Cordaro M. Crupi R. Esposito E. Petrosino S. (2017). Anti-inflammatory and neuroprotective effects of Co-UltraPEALut in a mouse model of vascular dementia. Front. Neurol. 8, 233. 10.3389/fneur.2017.00233 28634464 PMC5460147

[B68] Siracusa R. Paterniti I. Cordaro M. Crupi R. Bruschetta G. Campolo M. (2018). Neuroprotective effects of temsirolimus in animal models of Parkinson's disease. Mol. Neurobiol. 55 (3), 2403–2419. 10.1007/s12035-017-0496-4 28357809

[B69] Slemmer J. E. Shacka J. J. Sweeney M. I. Weber J. T. (2008). Antioxidants and free radical scavengers for the treatment of stroke, traumatic brain injury and aging. Curr. Med. Chem. 15 (4), 404–414. 10.2174/092986708783497337 18288995

[B70] Smith F. M. Raghupathi R. MacKinnon M. A. McIntosh T. K. Saatman K. E. Meaney D. F. (2000). TUNEL-positive staining of surface contusions after fatal head injury in man. Acta Neuropathol. 100 (5), 537–545. 10.1007/s004010000222 11045676

[B71] Stacchiotti A. Corsetti G. (2020). Natural compounds and autophagy: allies against neurodegeneration. Front. Cell. Dev. Biol. 8, 555409. 10.3389/fcell.2020.555409 33072744 PMC7536349

[B72] Stoica B. A. Faden A. I. (2010). Cell death mechanisms and modulation in traumatic brain injury. Neurotherapeutics 7 (1), 3–12. 10.1016/j.nurt.2009.10.023 20129492 PMC2841970

[B73] Villanueva-Paz M. Cotán D. Garrido-Maraver J. Oropesa-Ávila M. de la Mata M. Delgado-Pavón A. (2016). AMPK regulation of cell growth, apoptosis, autophagy, and bioenergetics. Exp. Suppl. 107, 45–71. 10.1007/978-3-319-43589-3_3 27812976

[B74] Wang P. Zhou X. Cui D. Ouyang T. Chen W. (2022). A single bout of exhaustive treadmill exercise increased AMPK activation associated with enhanced autophagy in mice skeletal muscle. Clin. Exp. Pharmacol. Physiol. 49 (4), 536–543. 10.1111/1440-1681.13632 35108422

[B75] Wang Y. J. Huang L. Q. Jiang C. Shen Y. (2013). Cloning and bioinformatics analysis of chorismate mutase gene from Salvia miltiorrhiza. Zhongguo Zhong Yao Za Zhi 38 (11), 1697–1702. 24010280

[B76] Wu J. Lipinski M. M. (2019). Autophagy in neurotrauma: good, bad, or dysregulated. Cells 8 (7), 693. 10.3390/cells8070693 31295858 PMC6678153

[B77] Ze Y. Hu R. Wang X. Sang X. Ze X. Li B. (2014). Neurotoxicity and gene-expressed profile in brain-injured mice caused by exposure to titanium dioxide nanoparticles. J. Biomed. Mater Res. A 102 (2), 470–478. 10.1002/jbm.a.34705 23533084

[B78] Zeng Z. Zhang Y. Jiang W. He L. Qu H. (2020). Modulation of autophagy in traumatic brain injury. J. Cell. Physiol. 235 (3), 1973–1985. 10.1002/jcp.29173 31512236

[B79] Zhang M. Shan H. Chang P. Wang T. Dong W. Chen X. (2014). Hydrogen sulfide offers neuroprotection on traumatic brain injury in parallel with reduced apoptosis and autophagy in mice. PLoS One 9 (1), e87241. 10.1371/journal.pone.0087241 24466346 PMC3900713

[B80] Zhang X. Chen Y. Jenkins L. W. Kochanek P. M. Clark R. S. B. (2005). Bench-to-bedside review: apoptosis/programmed cell death triggered by traumatic brain injury. Crit. Care 9 (1), 66–75. 10.1186/cc2950 15693986 PMC1065095

[B81] Zhao P. Zhou R. Zhu X. Y. Liu G. Zhao Y. P. Ma P. S. (2017). Neuroprotective effects of lycium barbarum polysaccharide on focal cerebral ischemic injury in mice. Neurochem. Res. 42 (10), 2798–2813. 10.1007/s11064-017-2293-x 28508173

