# Supplementary material for: Autophagy machinery plays an essential role in traumatic brain injury-induced apoptosis and its related behavioral abnormalities in mice: focus on Boswellia Sacra gum resin
Source: Front Physiol. 2024 Jan 5;14:1320960. doi: 10.3389/fphys.2023.1320960 (PMC10797063; doi:10.3389/fphys.2023.1320960)
Supplement: Supplementary file 1 [file DataSheet2.PDF]

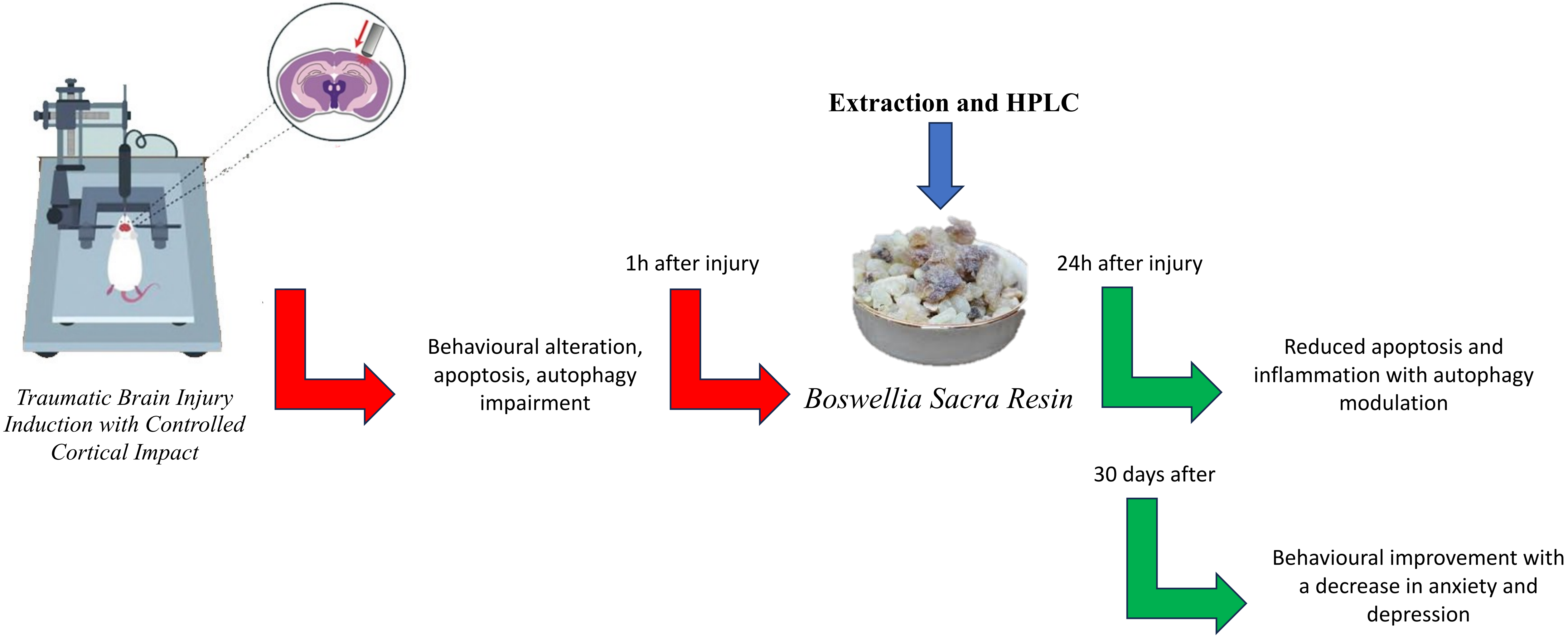
Extraction and HPLC
1h after injury
24h after injury
Behavioural alteration, apoptosis, autophagy impairment
Traumatic Brain Injury Induction with Controlled Cortical Impact
Boswellia Sacra Resin
Reduced apoptosis and inflammation with autophagy modulation
30 days after
Behavioural improvement with a decrease in anxiety and depression
