# Supplementary figures and images for: Autophagy machinery plays an essential role in traumatic brain injury-induced apoptosis and its related behavioral abnormalities in mice: focus on Boswellia Sacra gum resin
Source: Front Physiol. 2024 Jan 5;14:1320960. doi: 10.3389/fphys.2023.1320960 (PMC10797063; doi:10.3389/fphys.2023.1320960)

Bax  
23 kDa

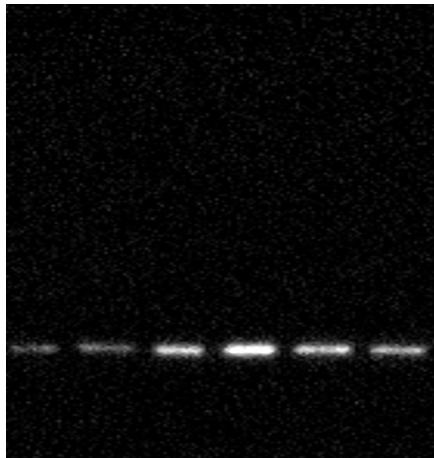

Bcl2  
29 kDa

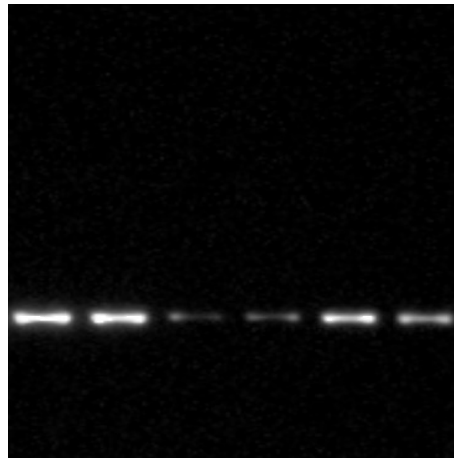

$\beta$ -Actin  
45 kDa

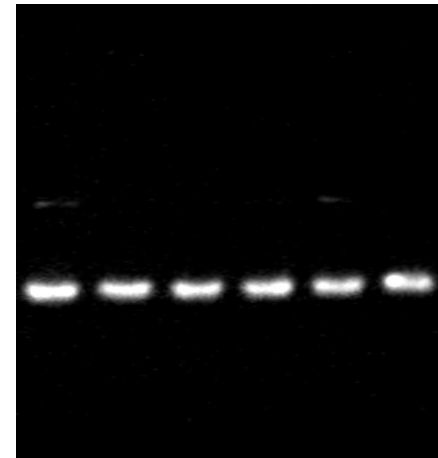

LC3  
15 kDa

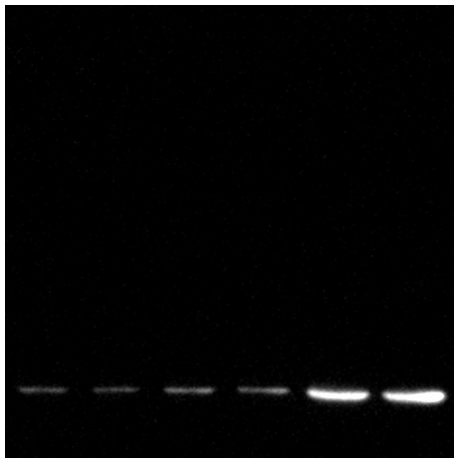

Beclin-1  
60 kDa

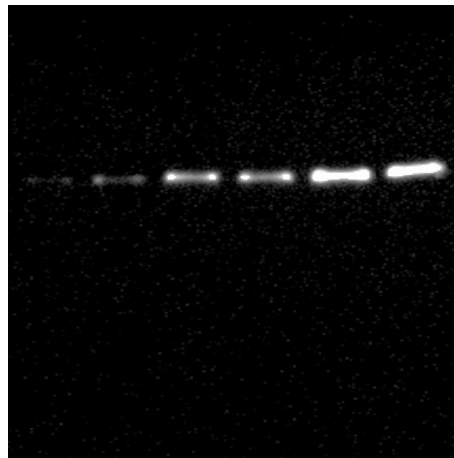

$\beta$ -Actin  
45 kDa

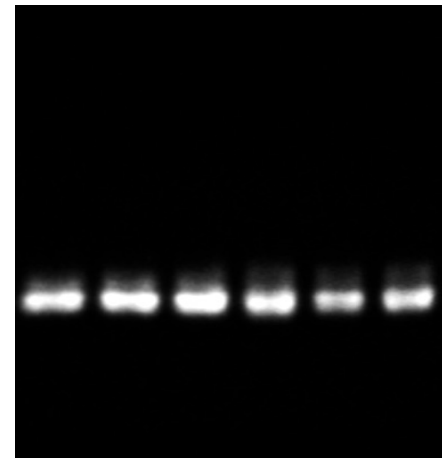

Supplement: Supplementary file 2 [file DataSheet3.PDF]

### *First set of experiment*

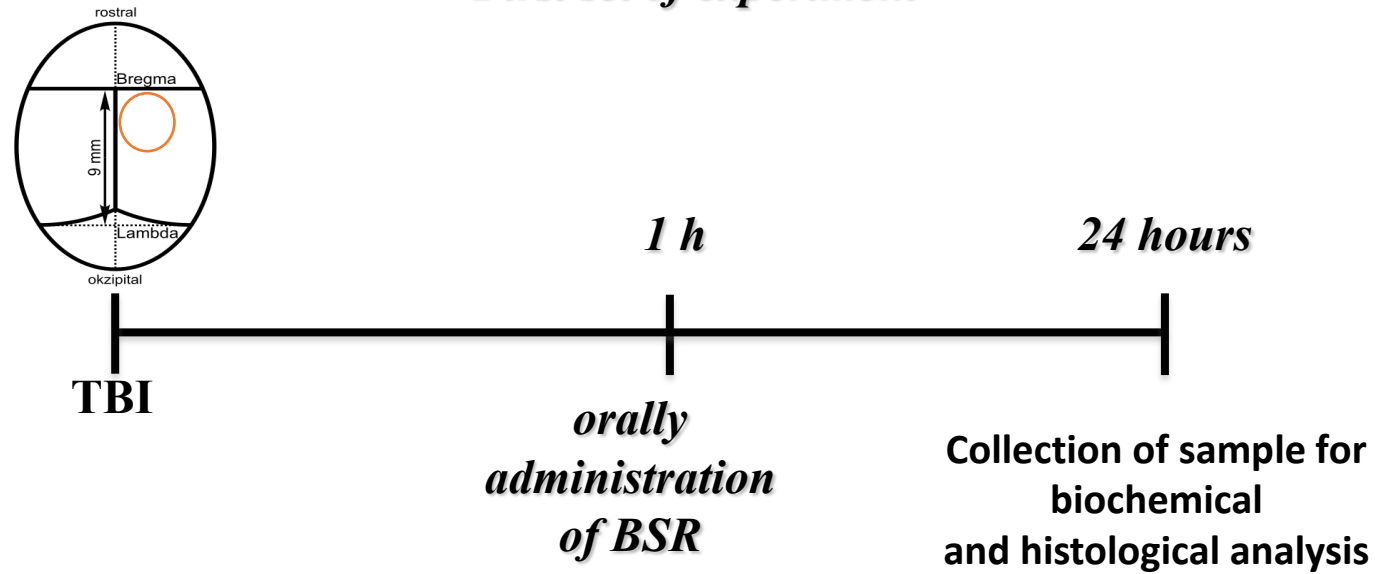

### *Second set of experiment*

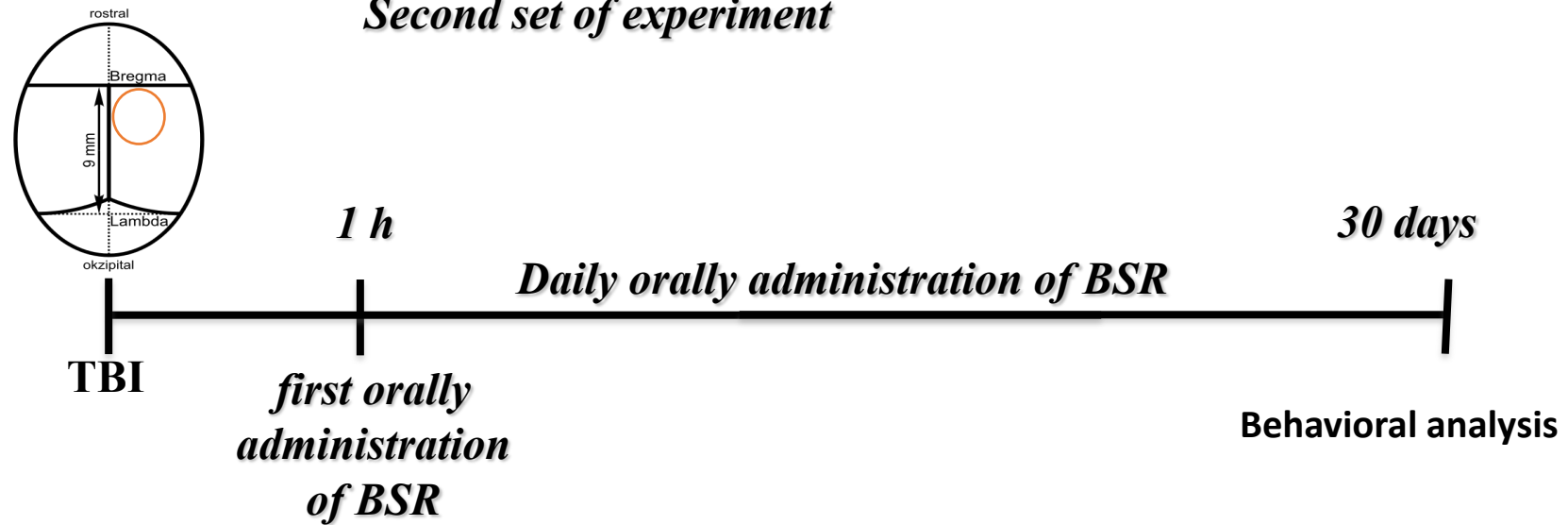

Supplement: Supplementary file 3 [file DataSheet1.PDF]
